# A mixed-methods study on healthcare workers’ perceptions of treatment adherence among HIV-TB co-infected patients in a multi-disease prevention policy context

**DOI:** 10.3389/fpubh.2025.1704215

**Published:** 2025-12-03

**Authors:** Ruili Bi, Lingwei Dou, Rong Pei, Chunnong Jike, Gang Yu, Ju Wang, Yifei Zheng

**Affiliations:** 1 School of Public Administration, China University of Geosciences, Wuhan, Hubei, China; 2 School of Public Health, Chengdu University of Traditional Chinese Medicine, Chengdu, Sichuan, China; 3 Warwick Business School, University of Warwick, Coventry, United Kingdom, China; 4 Liangshan Prefecture Center for Disease Control and Prevention, Xichang, Sichuan, China

**Keywords:** HIV-TB co-infection, treatment adherence, multi-disease prevention policy, healthcare workers, mixed-methods

## Abstract

**Background:**

Integrated service models aim to improve HIV–tuberculosis (TB) comorbidity management, yet little is known about how frontline providers perceive adherence challenges under China’s Integrated Prevention and Control of Four Diseases (IPC4D) policy. This mixed-methods study explored healthcare workers’ assessments of adherence, perceived barriers, and policy effects in Liangshan Prefecture—a high-burden, resource-limited, multi-ethnic region.

**Methods:**

Between May and June 2025, an online survey of 492 healthcare workers and 30 in-depth interviews were conducted. Quantitative data were analyzed using descriptive statistics and ordinal logistic regression [odds ratios (ORs), 95% confidence intervals (CIs)]; qualitative data underwent thematic analysis.

**Results:**

Overall, 64.0% of respondents rated patient adherence as “good” or “very good,” yet intermittent medication use (50.4%), unsupervised discontinuation (43.7%), and missed follow-ups (37.8%) remained common. Key perceived barriers included low health literacy (86.8%), regimen complexity (62.8%), side effects (61.2%), financial burden (59.8%), and limited family support (52.2%). Female respondents were less likely to report high adherence (OR = 0.57, 95% CI: 0.36–0.91), while clinicians (OR = 2.67, 95% CI: 1.35–5.31) and those in infectious disease departments (OR = 2.38, 95% CI: 1.23–4.64) reported more favorable assessments. Standardized adherence assessment correlated with lower reported adherence (OR = 0.16, 95% CI: 0.09–0.28), whereas institutional efforts to reduce financial burden were linked to higher adherence (OR = 1.78, 95% CI: 1.02–3.11). Qualitative findings highlighted persistent stigma, socioeconomic barriers, and mixed experiences with IPC4D implementation.

**Conclusion:**

Healthcare workers recognize IPC4D’s value in improving coordination and access but report enduring multilevel barriers. Strengthening policy impact requires standardized adherence monitoring, socioeconomic support, workforce development, and culturally sensitive patient education.

## 1 Introduction

Tuberculosis (TB) and human immunodeficiency virus (HIV) infection remain two of the most pressing public health challenges worldwide. According to the World Health Organization (WHO) Global Tuberculosis Report 2024, approximately 662,000 new TB cases in 2023 occurred among individuals living with HIV, and 161,000 HIV-positive individuals died from TB (1). TB continues to be the leading cause of death among people living with HIV (PLHIV), who are estimated to be 16 times more likely to develop active TB than HIV-negative individuals (2). This dual epidemic substantially increases clinical complexity and imposes a persistent burden on health systems across the globe.

China is among the countries with a high TB burden (3), with a particularly severe overlap of HIV and TB epidemics observed in the Liangshan Yi Autonomous Prefecture (Liangshan Prefecture), a remote ethnic minority region in the southwest (4, 5). This remote and resource-limited area is characterized by geographic isolation, underdeveloped primary healthcare infrastructure, and low health literacy, all of which exacerbate the challenges of managing HIV–TB co-infection (6).

Adherence to antiretroviral therapy and anti-TB treatment remains a decisive determinant of patient prognosis (7). Complex treatment regimens, adverse drug reactions, limited health literacy, and financial or transportation barriers frequently lead to treatment interruptions and inadequate follow-up (8–10). Social stigma and privacy concerns further undermine effective communication and trust between patients and healthcare providers (11, 12). Although integrated treatment models have demonstrated significant potential to reduce mortality, suboptimal adherence continues to represent a major barrier to effective disease control.

To address the complex challenges associated with managing multiple infectious diseases, China launched the “Integrated Prevention and Control of Four Diseases” (IPC4D) policy in 2021 in Liangshan Prefecture, a region with a particularly high burden of communicable diseases (13). This strategy integrates the screening, follow-up, and management of HIV/AIDS, tuberculosis, hepatitis C, and syphilis, with an emphasis on multi-sectoral collaboration, centralized coordination, and optimized resource allocation. The IPC4D policy is regarded as a key mechanism for strengthening primary healthcare integration, improving patient adherence, and enhancing health outcomes in resource-limited settings (14, 15). Nevertheless, empirical evidence remains scarce regarding how frontline healthcare workers perceive and respond to adherence-related challenges within this policy framework.

Frontline healthcare workers are critical actors in the implementation of multi-disease management strategies. They serve as the operational interface between national policies and real-world clinical practice. Their perceptions, experiences, and strategies provide valuable insights into the discrepancies between policy design and practical execution, especially in culturally diverse and resource-constrained settings.

This study investigates the implementation of the IPC4D policy using a mixed-methods design that combines quantitative and qualitative data. A structured survey was administered to 492 healthcare workers, complemented by in-depth interviews with 30 purposively selected participants. The study addresses four key research questions: (1) How do healthcare workers assess treatment adherence among patients co-infected with HIV and TB? (2) What barriers do they perceive as most influential in undermining adherence? (3) What intervention strategies have been adopted in clinical practice, and how effective are these strategies perceived to be? (4) How do healthcare workers view the contribution of the IPC4D policy to strengthening adherence management?

By integrating quantitative findings with qualitative insights, this study seeks to fill an important knowledge gap regarding healthcare workers’ perspectives on adherence under China’s multi-disease prevention policy. The findings aim to generate context-specific and actionable recommendations for optimizing adherence strategies in culturally diverse and resource-limited settings.

## 2 Methods

### 2.1 Study design and setting

This study employed a concurrent mixed-methods design that integrated structured questionnaire surveys with semi-structured, in-depth interviews to comprehensively examine healthcare workers’ observations, interpretations, and practical responses regarding treatment adherence among HIV–TB co-infected patients under the IPC4D policy. The study was conducted in Liangshan Yi Autonomous Prefecture, Sichuan Province, China—one of the regions with the highest HIV–TB co-infection burden and a pilot site for implementing the IPC4D policy, which integrates the prevention and management of HIV, TB, hepatitis C, and syphilis (16). The quantitative component aimed to capture broad patterns and determinants of healthcare workers’ perceptions, while the qualitative component sought to provide in-depth contextual explanations. Data integration occurred at the analysis and interpretation stages, allowing for triangulation and mutual enhancement of findings.

### 2.2 Participants and data collection

#### 2.2.1 Quantitative component (May 2025)

A cross-sectional structured online survey was administered in May 2025 to healthcare workers across Liangshan Prefecture. A multistage purposive sampling strategy was used to ensure representation from diverse institutional levels (county hospitals, township health centers, and ART clinics) and professional categories. Eligible participants were required to (1) be actively involved in HIV or TB services, including screening, diagnosis, treatment, or follow-up care; and (2) have at least 6 months of professional experience in their current position.

A total of 492 healthcare workers completed the survey. The sample included physicians, nurses, public health practitioners, and administrative staff. This diverse recruitment strategy aimed to enhance representativeness and minimize selection bias. Nevertheless, as participation was voluntary, potential self-selection bias cannot be fully excluded. All participants provided electronic informed consent prior to data collection.

#### 2.2.2 Qualitative component (June 2025)

To explore and explain patterns emerging from the survey data, semi-structured in-depth interviews were conducted in June 2025 with a purposive subsample of 30 healthcare workers, selected to reflect heterogeneity in professional roles, institutional levels, and geographic contexts (urban vs. rural). Participants included clinicians, nurses, and public health personnel from infectious disease departments, TB clinics, and community health facilities. Recruitment continued until data saturation was reached, i.e., when no new themes emerged from successive interviews.

Interviews were conducted face-to-face or via secure online platforms, depending on participants’ availability. Each interview lasted approximately 45–60 min and explored real-world adherence behaviors, perceived barriers, institutional responses, and implementation experiences related to the IPC4D policy. All interviews were audio-recorded with participants’ consent, transcribed verbatim, and anonymized to protect confidentiality.

### 2.3 Questionnaire design and measurement content

The structured questionnaire comprised both closed- and open-ended questions organized into four domains:

1. Sociodemographic and professional background;
2. Observations and assessments of treatment adherence among HIV–TB co-infected patients;
3. Perceptions of barriers and intervention measures;
4. Awareness, acceptance, and implementation experiences related to the IPC4D policy.

The adherence assessment used a 5-point Likert scale (1 = “very poor” to 5 = “very good”), complemented by multiple-choice and short-answer questions to capture nuanced feedback. To ensure content validity, the questionnaire was developed based on previous adherence and implementation studies and reviewed by a panel of five public health and infectious disease experts. A pilot test with 30 healthcare workers was conducted to refine item clarity and relevance. Internal consistency reliability was assessed using Cronbach’s alpha (*α* = 0.87), indicating good reliability.

### 2.4 Quantitative data analysis

Quantitative data were analyzed using IBM SPSS Statistics version 27.0. Descriptive statistics (frequencies, percentages, and means) were calculated for sociodemographic variables and adherence perceptions. Ordinal logistic regression was applied to examine factors associated with healthcare workers’ adherence assessments. Independent variables included gender, ethnicity, professional title, department, and the availability of institutional adherence tools and policy incentives (e.g., financial subsidies).

Before analysis, the proportional odds assumption was tested and met. Missing data (<5%) were handled using list wise deletion, as they were found to be missing completely at random (MCAR). Reference categories for categorical variables were set based on standard practice (e.g., male, Han ethnicity, general department). A two-tailed *p* < 0.05 was considered statistically significant.

### 2.5 Qualitative data analysis

The qualitative interview data were analyzed using thematic analysis, following the six-phase approach outlined by Braun and Clarke (17): (1) data familiarization; (2) initial coding; (3) theme development; (4) theme review; (5) theme definition and naming; and (6) report production. Two researchers independently coded the transcripts, and discrepancies were resolved through iterative discussion to achieve consensus. Intercoder reliability was assessed, achieving a Cohen’s kappa of 0.82, indicating substantial agreement. NVivo 12 software was used to facilitate coding and data organization.

Reflexivity was maintained throughout the analysis: researchers kept analytic memos to critically reflect on their positionality and potential influence on interpretation. Data saturation was achieved after 27 interviews, and the remaining three interviews confirmed thematic consistency. The final themes captured both systemic and individual-level factors influencing adherence, providing explanatory depth to the quantitative results.

## 3 Results

### 3.1 Participant demographics

A total of 492 healthcare workers participated in the study (Table 1). Most participants were female (74.4%) and aged 31–40 years (40.4%), indicating a relatively young and mid-career workforce. Nearly half identified as Han (47.6%) and 41.7% as Yi ethnicity, reflecting Liangshan’s ethnic diversity. The majority were married (81.7%) and held tertiary-level education, with 51.6% possessing a bachelor’s degree and 40.4% an associate degree.

**Table 1:** Descriptive characteristics of participating healthcare workers (*N =* 492).

Characteristic	Category	*n* (%)
Demographics		
Gender	Male/Female	126 (25.6)/366 (74.4)
Age group (years)	20–30/31–40/≥41	153 (31.1)/199 (40.4)/140 (28.5)
Ethnicity	Yi/Han/Other	205 (41.7)/234 (47.6)/53 (10.8)
Marital status	Married/Unmarried/Divorced or widowed	402 (81.7)/78 (15.9)/12 (2.4)
Education level	≤High school/Associate/Bachelor’s or above	38 (7.7)/199 (40.4)/255 (51.9)
Professional characteristics		
Professional background	Nursing/Clinical medicine/Other	227 (46.1)/181 (36.8)/84 (17.1)
Working years	≤5/6–10/11–20/>20	123 (25.0)/129 (26.2)/156 (31.7)/84 (17.1)
Type of institution	Township/County/Municipal/Other	245 (49.8)/108 (22.0)/89 (18.1)/50 (10.1)
Department	Public health/Infectious diseases/ART/Other	112 (22.8)/88 (17.9)/71 (14.4)/221 (44.9)
Professional role	Clinician/Nurse/Other	160 (32.5)/180 (36.6)/152 (30.9)
Professional title^1^	Junior/Intermediate/Senior/Other	217 (44.1)/93 (18.9)/28 (5.7)/154 (31.3)

Nearly half of participants worked at township hospitals, and 41.7% were Yi ethnicity, reflecting strong representation from grassroots healthcare providers. ^1^Professional title refers to the job rank assigned within the Chinese healthcare system.

Regarding professional background, nursing (46.1%) and clinical medicine (36.8%) were the most common fields. Approximately one-third of participants (31.7%) had 11–20 years of work experience, while another quarter (26.2%) had 6–10 years, indicating a balanced distribution of experience levels.

Institutionally, 49.8% worked in township hospitals, followed by county-level (22.0%) and municipal hospitals (18.1%), highlighting strong primary care representation. The main departments were public health (22.8%), infectious diseases (17.9%), and ART centers (14.4%). Nurses (36.6%) and clinicians (32.5%) formed the majority, with 44.1% holding junior titles and 18.9% intermediate ones, indicating that most participants were early- or mid-career providers engaged in frontline service delivery.

### 3.2 Healthcare workers’ observation and evaluation of adherence among HIV-TB co-infected patients

Quantitative findings from the survey are summarized in Table 2. Overall, healthcare workers reported a moderately positive view of treatment adherence among HIV–TB co-infected patients. Nearly two-thirds of respondents rated adherence as “good” (39.8%) or “very good” (24.2%), while about one-third considered it “average” (32.7%). Only a small fraction perceived adherence as “poor” or “very poor” (3.2% combined).

**Table 2:** Healthcare workers’ observation and evaluation of adherence among HIV–TB Co-infected patients (*N =* 492).

Variable	Category	*n* (%)
General evaluation of treatment adherence	Very good	119 (24.2)
	Good	196 (39.8)
	Average	161 (32.7)
	Poor/Very poor	16 (3.2)
Observed nonadherence behaviors	Intermittent medication	248 (50.4)
	Discontinuing medication without instruction	215 (43.7)
	Missing follow-ups	186 (37.8)
	Not cooperating with follow-up	174 (35.4)
Perceived reasons for poor adherence	Lack of health education and disease knowledge	427 (86.8)
	Complicated condition and treatment	309 (62.8)
	Side effects during treatment	301 (61.2)
	Economic burden	294 (59.8)
	Lack of family support	257 (52.2)
	Difficulties in transportation or access to care	242 (49.2)
	Insufficient communication/support from healthcare workers	139 (28.3)
Intervention measures taken	Yes	359 (72.8)
	No	133 (27.2)
Effectiveness of interventions	Significantly improved	182 (37.0)
	Slightly improved	240 (48.7)
	No improvement	70 (14.3)

The most frequently observed nonadherence behaviors included intermittent medication use (50.4%), discontinuation of treatment without medical consultation (43.7%), and missed follow-up appointments (37.8%). These findings suggest that irregular treatment and weak follow-up compliance remain key challenges in patient management.

Healthcare workers attributed poor adherence primarily to limited health literacy and insufficient disease knowledge (86.8%), followed by complex treatment regimens (62.8%), adverse side effects (61.2%), financial burden (59.8%), and lack of family support (52.2%). Additional barriers included transportation difficulties (49.2%) and inadequate communication or support from healthcare staff (28.3%). These perceived factors reflect both structural and patient-level constraints that hinder sustained adherence.

In response, 72.8% of healthcare workers reported implementing adherence-improvement interventions, such as enhanced counseling or community-based follow-up. Among them, 37.0% observed significant improvement, 48.7% reported slight improvement, and 14.3% found no evident change, indicating partial but uneven effectiveness of current intervention strategies.

### 3.3 Multivariate analysis of influencing factors on healthcare workers’ assessment of adherence among HIV-TB co-infected patients

An ordinal logistic regression analysis was conducted to identify factors influencing healthcare workers’ evaluations of treatment adherence among HIV-TB co-infected patients (Table 3).

**Table 3:** Ordered multinomial logistic regression analysis of factors associated with healthcare workers’ evaluation of treatment adherence among HIV-TB co-infected patients (*N =* 492).

Variable	*β*	SE	Wald *χ*^2^	*p*-value	OR (95% CI)
Gender (Ref: Male) – Female	−0.554	0.237	5.451	0.02	0.57 (0.36, 0.91)
Department (Ref: Others) – Infectious Diseases	0.869	0.34	6.542	0.011	2.38 (1.23, 4.64)
Position (Ref: Others) – Clinician	0.983	0.35	7.887	0.005	2.67 (1.35, 5.31)
Position (Ref: ART Center Director) – Clinician	1.495	0.538	7.726	0.005	4.46 (1.55, 12.81)
Main disease type encountered (Ref: HIV, TB, HCV, Syphilis) – HIV	−0.53	0.218	5.94	0.015	0.59 (0.38, 0.90)
Institutional adherence assessment procedures (Ref: None) – Yes	−1.813	0.277	42.934	<0.001	0.16 (0.09, 0.28)

Female healthcare workers were significantly less likely than males to rate patients as adherent (OR = 0.57, 95% CI: 0.36–0.91). Conversely, those working in infectious disease departments were over twice as likely to report higher adherence levels compared to other departments (OR = 2.38, 95% CI: 1.23–4.64). Clinicians showed greater odds of providing favorable adherence evaluations compared with other positions such as nurses or pharmacists (OR = 2.67, 95% CI: 1.35–5.31), and particularly when compared with ART center directors (OR = 4.46, 95% CI: 1.55–12.81).

Healthcare workers primarily treating HIV-only patients were less likely to report high adherence than those managing multiple diseases such as HIV, TB, and HCV (OR = 0.59, 95% CI: 0.38–0.90), suggesting that broader clinical exposure may enhance confidence in patients’ treatment behavior.

Institutional factors also played a key role. Workers in institutions with standardized adherence assessment procedures were significantly less likely to report good adherence (OR = 0.16, 95% CI: 0.09–0.28), possibly reflecting heightened vigilance in structured evaluation systems. In contrast, healthcare workers in institutions that implemented measures to reduce patients’ financial burden—such as subsidies or fee reductions—were more likely to report higher adherence levels (OR = 1.78, 95% CI: 1.02–3.11), suggesting that perceived economic support may positively influence adherence evaluations.

### 3.4 Attribution analysis of poor adherence among HIV-TB co-infected patients by healthcare workers

Drawing on semi-structured interviews with 30 healthcare workers, a thematic content analysis identified five major themes underlying poor treatment adherence among HIV–TB co-infected patients (Table 4). These themes highlight the interplay between individual, social, institutional, and contextual barriers that shape patients’ adherence behavior.

**Table 4:** Healthcare workers’ attributions for poor treatment adherence among HIV-TB co-infected patients.

Higher-order themes	Sub-themes
Individual-level factors	Lack of disease knowledge and health literacy
	Fear of side effects and low treatment confidence
	Privacy concerns and disease-related stigma
Lack of social support	Insufficient family support
	Weak community support
Structural and institutional factors	Inadequate communication and support from healthcare workers
	Complex treatment procedures and long visits
	Lack of adherence assessment and follow-up mechanisms
Economic and accessibility barriers	Heavy medical financial burden
	Inconvenient transportation and limited access to care
	High cost of work absence
Disease-specific factors	Comorbidity and complexity of conditions
	Multiple medications and poor medication adherence

**Individual-level factors** were the most frequently mentioned barriers. Many healthcare workers emphasized that patients’ limited disease knowledge and low health literacy hindered understanding of treatment importance. Fear of medication side effects and low treatment confidence often led to premature discontinuation. Privacy concerns and HIV/TB-related stigma further discouraged consistent medication-taking, especially in small communities where confidentiality was difficult to maintain.

**Lack of social support** was another recurring explanation. Inadequate family care and weak community engagement were perceived to reduce patients’ motivation and ability to sustain treatment. Respondents from rural and mountainous areas noted that many patients lived alone or had migrated family members, leaving them with limited emotional or logistical support.

At the **structural and institutional level**, several respondents cited limited provider–patient communication, long waiting times, and complex procedures as key system-level barriers. The absence of standardized adherence assessment tools or follow-up mechanisms was described as a “blind spot” in routine service delivery, making it difficult to identify and support patients at risk of nonadherence.

**Economic and accessibility barriers** were particularly prominent in under-resourced areas. High medical expenses, long travel distances to clinics, and income loss due to treatment-related work absences were perceived as major deterrents to adherence. These challenges were seen as structural constraints rather than individual patient failures.

Finally, **disease-specific factors** compounded these difficulties. The dual burden of HIV and TB, frequent comorbidities, and complex medication regimens contributed to confusion and unintentional nonadherence. Several healthcare workers described patients struggling with pill burden and medication timing, suggesting that the biological complexity of co-infection itself presents unique adherence challenges.

Together, these findings reveal that poor treatment adherence is shaped by interlocking factors across multiple levels, many of which reflect broader structural and institutional contexts rather than purely individual behavior.

### 3.5 Healthcare workers’ perceptions and evaluation of the IPC4D policy

This section integrates quantitative survey data and qualitative interviews to explore healthcare workers’ awareness, perceptions, and implementation experiences regarding the Integrated Prevention and Control of Four Diseases (IPC4D) policy. Overall, respondents demonstrated strong policy recognition and perceived substantial benefits in patient outcomes, resource coordination, and service accessibility, although persistent challenges were also reported. The main findings are summarized in Table 5.

**Table 5:** Healthcare workers’ perceptions and evaluation of the integrated prevention and control of four diseases policy: integration of survey and interview findings.

Higher-level themes	Subthemes	Survey-based evidence	Illustrative interview quotes
Policy awareness and implementation	Awareness of the Policy	95.7% of respondents reported being familiar with the policy	“I’ve attended policy meetings and participated in the implementation.” (P07, male, township hospital physician)
	Implementation in Facilities	96.1% confirmed policy implementation in their facility	“We’ve already fully rolled out the policy in our department.” (P12, male, ART center director)
Perceived health benefits	Improved Health Outcomes	52.9% of respondents reported significant health improvements, while 43.7% reported moderate improvements	“The treatment for co-infections is smoother now, and the outcomes are clearly better.” (P15, male, County hospital doctor)
	Enhanced Treatment Adherence	48.4% of respondents observed significant, and 46.3% observed moderate, improvement in adherence	“Centralized management made patients more cooperative with their medications.” (P19, female, primary care nurse)
Improved service accessibility	Access in Remote and Vulnerable Populations	95.5% of participants agreed that access to healthcare had improved	“Even in rural villages, people can access the same screening. They feel more valued.” (P09, female, township doctor)
Positive implementation outcomes	Resource Integration and Efficiency Gains	Many respondents highlighted “better resource integration and improved efficiency”	“Combining the four diseases streamlined screening and interventions, saving repeated efforts.” (P03, female, township hospital doctor)
	Enhanced Interdepartmental Collaboration	Interviewees repeatedly noted “more effective interdepartmental communication”	“The policy brought us together with CDC and pharmacy for joint meetings and training.” (P08, male, public health officer, county hospital)
Implementation challenges	Limited Resources and Capacity	Some participants cited staff shortages and limited capacity	“The policy is good, but in remote villages, we are understaffed and overloaded.” (P16, female, Village doctor)
	Inconsistent Execution	Some regions showed weaker enforcement	“Some areas have implemented it well, but others still need stronger oversight.” (P02, male, CDC staff)

#### 3.5.1 Theme 1: policy awareness and implementation (mentioned by 28/30 participants, 93%)

Most healthcare workers exhibited high awareness and active engagement in the IPC4D policy rollout. Quantitatively, 95.7% of respondents reported being familiar with the policy, and 96.1% confirmed its implementation in their facilities.

“I’ve attended policy meetings and participated in the implementation.” (P07, male, township hospital physician)

“We’ve already fully rolled out the policy in our department.” (P12, male, ART center director)

These results indicate strong top-down dissemination and local institutional engagement, aligning with prior studies emphasizing the effectiveness of vertical health governance structures in China’s county-level public health systems.

#### 3.5.2 Theme 2: perceived health benefits (mentioned by 24/30 participants, 80%)

A majority of healthcare workers perceived noticeable improvements in patient outcomes and treatment adherence following the policy’s implementation. Specifically, 52.9% of respondents reported significant health improvements among patients, while 43.7% reported moderate improvements. In addition, 48.4% of respondents observed significant, and 46.3% observed moderate, improvement in adherence.

“The treatment for co-infections is smoother now, and the outcomes are clearly better.” (P15, male, county hospital doctor)

“Centralized management made patients more cooperative with their medications.” (P19, female, primary care nurse)

Healthcare workers attributed these perceived gains to improved coordination of care, integrated follow-up mechanisms, and clearer treatment pathways. These findings echo previous literature showing that integrated management frameworks can enhance both clinical efficiency and patient adherence in multi-disease contexts.

#### 3.5.3 Theme 3: improved service accessibility (mentioned by 22/30 participants, 73%)

Healthcare workers widely recognized that the IPC4D policy had improved healthcare accessibility, especially in remote or vulnerable populations. Survey data indicated that 95.5% of participants agreed that access to healthcare had improved.

“Even in rural villages, people can access the same screening. They feel more valued.” (P09, female, township doctor)

“Before, many patients had to travel far for check-ups. Now the screening comes to them.” (P03, male, village clinician)

These findings reflect the policy’s contribution to reducing geographic and financial barriers to care, promoting health equity across diverse regions, consistent with prior studies on rural HIV-TB integration programs in low-resource settings.

#### 3.5.4 Theme 4: positive implementation outcomes (mentioned by 20/30 participants, 67%)

Respondents emphasized that IPC4D enhanced resource integration and interdepartmental collaboration. Many healthcare workers described efficiency gains due to shared screening, diagnostic, and reporting mechanisms.

“Combining the four diseases streamlined screening and interventions, saving repeated efforts.” (P03, female, township hospital doctor)

“The policy brought us together with CDC and pharmacy for joint meetings and training.” (P08, male, public health officer, county hospital)

These findings demonstrate that IPC4D fostered synergy among clinical, public health, and pharmaceutical units, reinforcing intersectoral collaboration and optimizing resource utilization. Such integration aligns with international evidence suggesting that horizontal coordination mechanisms improve service delivery efficiency.

#### 3.5.5 Theme 5: implementation challenges (mentioned by 26/30 participants, 87%)

Despite strong policy support, healthcare workers highlighted persistent challenges, particularly in under-resourced rural areas. Commonly reported issues included insufficient staffing, limited capacity, and regional disparities in policy execution.

“The policy is good, but in remote villages, we’re understaffed and overloaded.” (P16, female, village doctor)

“Some areas have implemented it well, but others still need stronger oversight.” (P02, male, CDC staff)

These challenges reflect structural limitations in local health systems, particularly workforce shortages and inconsistent supervision. The uneven implementation observed across regions underscores the need for tailored capacity-building strategies to ensure equitable and sustainable policy outcomes.

## 4 Discussion

This mixed-methods study provides one of the first empirical assessments of healthcare workers’ perceptions, experiences, and evaluations regarding treatment adherence among HIV–TB co-infected patients under China’s Integrated Prevention and Control of Four Diseases (IPC4D) policy. By triangulating large-scale quantitative data with in-depth qualitative insights, this study identifies the multilevel barriers shaping adherence behavior and highlights the potential and limitations of integrated disease control in a resource-limited, multi-ethnic region.

### 4.1 Overview of main findings and interpretation

Consistent with prior research (18–20). over 60% of healthcare workers rated patient adherence as “good” or “very good,” yet nearly one-third simultaneously reported persistent nonadherence. This paradox highlights the coexistence of improvement and ongoing barriers in adherence management. Intermittent medication use, missed follow-up visits, and treatment interruptions remain the most frequently reported issues, suggesting that behavioral continuity—rather than initiation—is the key weakness in sustaining adherence. Patient adherence remains a significant barrier to achieving effective treatment outcomes and controlling disease in the context of HIV-TB co-infection (21–23).

A particularly noteworthy finding is the adherence rating paradox. Several explanations may account for this contradiction. First, differences in evaluation standards exist among healthcare workers—clinicians often assess adherence by clinical outcomes, while nurses and CDC staff emphasize daily medication behavior. Second, patient subgroup variation (urban vs. rural, Han vs. Yi ethnicity) may contribute to inconsistent adherence patterns. Third, social desirability bias may lead to over reporting of positive outcomes in surveys relative to interviews. Finally, varying definitions of “good adherence,” ranging from general cooperation to full regimen compliance, create subjective inconsistencies. Together, these factors indicate that healthcare workers’ perceptions are influenced by contextual and evaluative diversity, cautioning against simplistic interpretations of overall adherence rates. Future evaluations should adopt standardized, multi-source adherence metrics that integrate objective and perceptual data.

### 4.2 Determinants of adherence and institutional influences

Multivariate results underscore that adherence evaluations are shaped not only by individual behavior but also by institutional capacity and organizational culture. Healthcare workers in infectious disease departments or those with greater IPC4D implementation experience tended to report higher adherence, reflecting stronger clinical expertise and closer patient management. In contrast, facilities with standardized adherence assessments reported lower adherence ratings—likely reflecting stricter monitoring rather than poorer performance, a finding consistent with studies showing that structured audits often uncover hidden adherence issues (24, 25).

Conversely, institutions that adopted financial support measures—such as medical subsidies or travel reimbursement—reported significantly better adherence outcomes (OR = 1.78, 95% CI: 1.02–3.11). These results highlight the importance of economic relief and institutional support systems in sustaining long-term adherence. Together, these findings affirm that adherence is a systemic outcome of organizational structure, resource distribution, and professional engagement rather than a purely individual behavioral issue.

### 4.3 Barriers and contextual challenges

Qualitative findings revealed a web of interconnected barriers spanning individual, social, and structural domains. At the individual level, low health literacy, treatment fatigue, stigma, and fear of side effects—particularly among ethnic minority groups—undermined adherence. At the social level, weak family and community support further reduced patient motivation. Structurally, long travel distances, poor transportation, limited staff, and fragmented follow-up systems hindered treatment continuity.

These barriers were especially pronounced in rural and minority communities, where economic hardship and linguistic barriers constrained care access. Moreover, healthcare workers reported institutional overload, with heavy workloads and limited supervision capacity, which restricted their ability to deliver patient counseling and community-based follow-up. These findings resonate with socioecological models of adherence, underscoring that sustainable adherence requires simultaneous action on individual behavior, social support, and systemic infrastructure (24–27).

Additionally, healthcare workers identified institutional barriers that further undermine patients’ willingness to continue their treatment. These barriers include complex service procedures, inadequate follow-up systems, and limited time available for patient communication. This understanding of the situation is consistent with systematic reviews that highlight the multifaceted obstacles impacting treatment adherence in resource-limited settings (28–30).

### 4.4 Policy implications of the IPC4D framework

Healthcare workers widely viewed the IPC4D policy as a transformative framework for integrating service delivery, improving care coordination, and enhancing patient outcomes. Quantitative data showed that 52.9% of healthcare workers observed significant and 43.7% moderate improvements in patient health, while 48.4% reported significant and 46.3% moderate improvements in adherence after policy rollout. These perceived gains stemmed from integrated screening, unified data systems, and joint management processes that reduced service duplication and enhanced accessibility—especially in remote areas (31–33).

These local observations align with WHO recommendations that emphasize collaborative TB–HIV activities, people-centred and integrated services, and strengthening governance and monitoring for comorbidities as core components of TB/HIV control strategies. WHO’s operational guidance and consolidated TB modules recommend integrated screening, rapid ART initiation for people with TB/HIV, and strengthening of monitoring systems to improve outcomes for co-infected patients (34, 35).

Recent empirical syntheses reinforce these principles: systematic reviews and implementation studies from low- and middle-income settings indicate that full or partial integration of HIV and TB services—when paired with appropriate staffing, data systems and patient-centred supports—can improve retention in care and some clinical outcomes, although effects on hard endpoints vary by context and implementation fidelity. These reviews also highlight that integration alone is insufficient without investments in workforce capacity, digital data systems, and targeted socioeconomic supports—findings that mirror our study’s identification of persistent bottlenecks (staff shortages, uneven supervision, data-sharing gaps) (8).

Finally, recent programmatic evidence and WHO guidance increasingly emphasize differentiated service delivery (DSD) and the prudent use of digital adherence technologies (DATs) as complements to integrated models—especially to maintain continuity of care and to reach remote populations. While DATs (SMS, electronic pillboxes, remote follow-up) have shown promise for improving adherence in some settings, their effectiveness is heterogeneous and context-dependent; they should therefore be deployed alongside social and economic enablers rather than as standalone fixes (36).

### 4.5 Theoretical and practical implications

The findings support a systems-oriented and socioecological interpretation of adherence behavior: structural enablers (financing, transport, staffing), organizational processes (standardized monitoring, interdepartmental coordination), and individual psychosocial factors jointly determine outcomes. This multi-level perspective is consistent with WHO’s call to coordinate planning, governance, and people-centred services for managing comorbidities, and with recent evidence advocating integrated, differentiated, and context-sensitive delivery models (37).

Practically, our results suggest three complementary operational priorities that reflect both WHO guidance and recent empirical findings: (1) scale up integrated, one-stop services for HIV/TB with clear referral and data-sharing protocols; (2) institutionalize targeted socioeconomic supports (subsidies, transport reimbursement) and community-based follow-up to address the non-clinical determinants of nonadherence; and (3) adopt DSD approaches and selectively deploy DATs as part of a package that includes human support and monitoring, with built-in evaluation to assess impact and equity. These priorities align with WHO operational recommendations and the recent literature showing that combined health-system and social interventions yield better retention and adherence than either approach alone (38).

### 4.6 Limitations

This study has several limitations. First, its cross-sectional design limits causal interpretation between policy implementation and observed adherence changes. Second, the reliance on healthcare workers’ perceptions may introduce subjectivity and social desirability bias, as objective clinical data (e.g., viral load, TB conversion) were unavailable. Third, the study focuses on Liangshan Prefecture, and findings may not generalize to other settings with different sociocultural profiles. Finally, patient voices were not directly included; future research should incorporate patient narratives and longitudinal data to triangulate adherence assessments and capture dynamic behavioral patterns.

## 5 Conclusion

This study provides an integrated assessment of healthcare workers’ experiences with the IPC4D policy in Liangshan Prefecture, China, revealing both substantial progress and persistent challenges in the integrated management of HIV–TB co-infection within multi-ethnic, resource-constrained settings. Healthcare workers widely recognized the policy’s role in improving coordination, service accessibility, and patient outcomes, yet also highlighted enduring systemic barriers such as workforce shortages, financial constraints, and uneven implementation across regions. The coexistence of high adherence ratings and continuing adherence challenges underscores the context-dependent and subjective nature of adherence assessment, influenced by differing evaluation standards, subgroup variations, and potential reporting biases. These findings emphasize the necessity of establishing standardized, multi-source adherence monitoring systems that integrate patient perspectives with objective indicators.

Moving forward, strengthening the IPC4D framework will require sustained system-level investment and adaptive governance. Key priorities include expanding patient-centered education and stigma reduction—particularly among ethnic minority groups—enhancing financial and logistical support to mitigate treatment barriers, and institutionalizing digital monitoring and standardized adherence evaluation tools. Continued workforce development, intersectoral collaboration, and community participation are equally essential to ensure policy responsiveness and sustainability. Ultimately, the IPC4D model represents a promising and equity-oriented approach to comorbidity management in low-resource contexts, yet its long-term success depends on transforming structural integration into an enduring, people-centered practice that advances global health equity.

## Data Availability

The original contributions presented in the study are included in the article/supplementary material, further inquiries can be directed to the corresponding author.

## References

[1] WHO. Global tuberculosis report 2024. Geneva: WHO (2024).

[2] WHO. Tuberculosis. Geneva: WHO (2025).

[3] Yang N He J Li J Zhong Y Song Y Chen C. Predictors of death among TB/HIV co-infected patients on tuberculosis treatment in Sichuan, China: a retrospective cohort study. Medicine. (2023) 102:e32811. doi: 10.1097/md.0000000000032811, 36749231 PMC9901956

[4] China, Yearbook HS. (2025). China health statistics yearbook. Available online at: https://www.nhc.gov.cn/mohwsbwstjxxzx/tjtjnj/202501/8193a8edda0f49df80eb5a8ef5e2547c.shtml (Accessed August 25, 2025).

[5] Li J He J Li T Li Y Gao W Zhong Y . 6-month regimen of isoniazid prevention therapy for tuberculosis among people living with human immunodeficiency virus in minority areas of China: a 3-year prospective cohort study. BMJ Open Respir Res. (2024) 11:801. doi: 10.1136/bmjresp-2024-002801, 39721747 PMC11683910

[6] Liao R Hu L Yu J Chen Y Chen M Yan J . Association between TB delay and TB treatment outcomes in HIV-TB co-infected patients: a study based on the multilevel propensity score method. BMC Infect Dis. (2024) 24:457. doi: 10.1186/s12879-024-09328-7, 38689228 PMC11061920

[7] Moges S Lajore BA. Mortality and associated factors among patients with TB-HIV co-infection in Ethiopia: a systematic review and meta-analysis. BMC Infect Dis. (2024) 24:773. doi: 10.1186/s12879-024-09683-5, 39095740 PMC11295522

[8] Dlatu N Longo-Mbenza B Apalata T. Models of integration of TB and HIV services and factors associated with perceived quality of TB-HIV integrated service delivery in O. R Tambo District, South Africa. BMC Health Serv Res. (2023) 23:804. doi: 10.1186/s12913-023-09748-2, 37501061 PMC10375732

[9] Addo J Pearce D Metcalf M Lundquist C Thomas G Barros-Aguirre D . Living with tuberculosis: a qualitative study of patients' experiences with disease and treatment. BMC Public Health. (2022) 22:1717. doi: 10.1186/s12889-022-14115-7, 36085073 PMC9462890

[10] Anku PJ Amo-Adjei J Doku DT Kumi-Kyereme A. Integration of tuberculosis and HIV services: exploring the perspectives of co-infected patients in Ghana. Glob Public Health. (2018) 13:1192–203. doi: 10.1080/17441692.2017.1385823 28984493

[11] Kipp AM Pungrassami P Stewart PW Chongsuvivatwong V Strauss RP Van Rie A. Study of tuberculosis and AIDS stigma as barriers to tuberculosis treatment adherence using validated stigma scales. Int J Tuberc Lung Dis. (2011) 15:1540–5. doi: 10.5588/ijtld.10.0273, 22008770 PMC3753693

[12] Canetti D Riccardi N Martini M Villa S Di Biagio A Codecasa L . HIV and tuberculosis: the paradox of dual illnesses and the challenges of their fighting in the history. Tuberculosis. (2020) 122:101921. doi: 10.1016/j.tube.2020.101921, 32501257

[13] Liangshan Daily. (2025). Policy interpretation: Implementation plan for phase II of the HIV/AIDS control strategy in Liangshan prefecture, China. Xichang, China: Liangshan Daily Press.

[14] China CDC. (2024). Guiding HIV prevention and advancing integrated control of four diseases: 2022 Liangshan workstation meeting held in Xichang City, Liangshan prefecture. Beijing, China: Chinese Center for Disease Control and Prevention.

[15] Xichang People’s Hospital. (2024). “Prevention of four diseases, elimination of three diseases” - Xichang people's hospital launches thematic health education publicity activities. Xichang, China: Xichang People’s Hospital.

[16] Liang X Chen M Wu G Zhong J. (2025). Sustained efforts in Liangshan Yi autonomous prefecture to ensure the effective and orderly implementation of the second phase of the HIV prevention initiative. Xichang, China: Liangshan Yi Autonomous Prefecture Government Information Office.

[17] Braun V Clarke V. What can "thematic analysis" offer health and wellbeing researchers? Int J Qual Stud Health Well-being. (2014) 9:26152. doi: 10.3402/qhw.v9.26152, 25326092 PMC4201665

[18] Legido-Quigley H Montgomery CM Khan P Atun R Fakoya A Getahun H. Integrating tuberculosis and HIV services in low- and middle-income countries: a systematic review. Trop Med Int Health. (2013) 18:199–211. doi: 10.1111/tmi.12029, Grant AD 23217030

[19] Laycock K Technau KG Lelo P Jantarabenjakul W Yonaba C Pinto J . Tuberculosis diagnosis, treatment, and prevention Services for Children Living with HIV in low- and middle-income countries: a multiregional site survey. J Pediatric Infect Dis Soc. (2025) 14:050. doi: 10.1093/jpids/piaf050, 40425522 PMC12257623

[20] Seunanden TC Ngwenya N Seeley J. Experiences and perceptions on antiretroviral therapy adherence and non-adherence: a scoping review of young people living with HIV in sub-Saharan Africa. BMC Public Health. (2025) 25:1450. doi: 10.1186/s12889-025-22579-6, 40247282 PMC12004696

[21] Olivier C Luies L. WHO goals and beyond: managing HIV/TB co-infection in South Africa. SN Compr Clin Med. (2023) 5:251. doi: 10.1007/s42399-023-01568-z

[22] Maseko AF Silumbwe A Maritim P Munakampe MN Chirambo GB Jacobs C . Factors that shape the integration of HIV and TB services in Zomba District, Malawi. BMC Health Serv Res. (2025) 25:213. doi: 10.1186/s12913-025-12367-8, 39915793 PMC11800521

[23] Patel A Pundkar A Agarwal A Gadkari C Nagpal AK Kuttan N. A comprehensive review of HIV-associated tuberculosis: clinical challenges and advances in management. Cureus 2024;16:e68784. doi: 10.7759/cureus.68784. PMID 39371702. 39371702 PMC11456262

[24] Azwar M Widiastuty L Setialaksana W. Social stigma, family support, and healthcare access as determinants of substance use among transgender populations: a quantitative study in South Sulawesi, Indonesia. Al-Sihah Public Health Sci J. (2025) 22:46–56. doi: 10.24252/al-sihah.v17i1.55863

[25] Wouters E Sommerland N Masquillier C Rau A Engelbrecht M Van Rensburg AJ . Unpacking the dynamics of double stigma: how the HIV-TB co-epidemic alters TB stigma and its management among healthcare workers. BMC Infect Dis. (2020) 20:4816. doi: 10.1186/s12879-020-4816-3, 32028895 PMC7006097

[26] Foster I Biewer A Vanqa N Makanda G Tisile P Hayward SE . This is an illness. No one is supposed to be treated badly: community-based stigma assessments in South Africa to inform TB stigma intervention design. Res Sq. (2023) 22:733. doi: 10.21203/rs.3.rs-3716733/v1, 38919729 PMC11194205

[27] Wouters E van Rensburg AJ Engelbrecht M Buffel V Campbell L Sommerland N . How the 'HIV/TB co-epidemic-HIV stigma-TB stigma' syndemic impacts on the use of occupational health services for TB in south African hospitals: a structural equation modelling analysis of the baseline data from the HaTSaH study (cluster RCT). BMJ Open. (2022) 12:e045477. doi: 10.1136/bmjopen-2020-045477, 35383052 PMC8984004

[28] Naidoo K Gengiah S Singh S Stillo J Padayatchi N. Quality of TB care among people living with HIV: gaps and solutions. J. Clin. Tuberculosis Mycobacterial Dis. (2019) 17:100122. doi: 10.1016/j.jctube.2019.100122, 31788564 PMC6880007

[29] Sazali MF Rahim S Mohammad AH Kadir F Payus AO Avoi R . Improving tuberculosis medication adherence: the potential of integrating digital technology and health belief model. Tuberc Respir Dis. (2023) 86:82–93. doi: 10.4046/trd.2022.0148, 36597583 PMC10073608

[30] Choi R Jeong B-H Koh W-J Lee S-Y. Recommendations for optimizing tuberculosis treatment: therapeutic drug monitoring, pharmacogenetics, and nutritional status considerations. Ann Lab Med. (2017) 37:97–107. doi: 10.3343/alm.2017.37.2.97, 28028995 PMC5204003

[31] Dlatu N Oladimeji KE Apalata T. Voices from the patients: a qualitative study of the integration of tuberculosis, human immunodeficiency virus and primary healthcare services in O.R. Tambo district, eastern cape, South Africa. Infect Dis Rep. (2023) 15:158–70. doi: 10.3390/idr15020017, 36960969 PMC10037593

[32] Enane LA Eby J Arscott-Mills T Argabright S Caiphus C Kgwaadira B . TB and TB-HIV care for adolescents and young adults. Int J Tuberc Lung Dis. (2020) 24:240–9. doi: 10.5588/ijtld.19.0416, 32127110 PMC7307717

[33] Daftary A Padayatchi N. Integrating patients' perspectives into integrated tuberculosis-human immunodeficiency virus health care. Int J Tuberc Lung Dis. (2013) 17:546–51. doi: 10.5588/ijtld.12.0714, 23407149 PMC3665901

[34] WHO. (2024). Recommendations in the WHO consolidated guidelines on tuberculosis: Tuberculosis preventive treatment, second edition (2024) and in the previous edition (2020). Available online at: https://tbksp.who.int/en/node/44 (accessed October 15, 2025).

[35] Bulstra CA Hontelez JAC Otto M Stepanova A Lamontagne E Yakusik A . Integrating HIV services and other health services: a systematic review and meta-analysis. PLoS Med. (2021) 18:e1003836. doi: 10.1371/journal.pmed.1003836, 34752477 PMC8577772

[36] Ehrenkranz P Grimsrud A Holmes CB Preko P Rabkin M. Expanding the vision for differentiated service delivery: a call for more inclusive and truly patient-centered Care for People Living with HIV. JAIDSd. (2021) 86:147–52. doi: 10.1097/QAI.0000000000002549 PMC7803437 33136818

[37] WHO. (2025). Treatment of DS-TB in people living with HIV. Available online at: https://tbksp.who.int/en/node/3023?utm_source=chatgpt.com (accessed October 10, 2025).

[38] WHO. (2020). WHO consolidated guidelines on tuberculosis. Available online at: https://www.who.int/publications/i/item/9789240001503 (accessed October 16, 2025).

