# Correction: A mixed-methods study on healthcare workers' perceptions of treatment adherence among HIV-TB co-infected patients in a multi-disease prevention policy context

**DOI:** 10.3389/fpubh.2025.1761803

**Published:** 2026-01-07

**Authors:** Ruili Bi, Lingwei Dou, Rong Pei, Chunnong Jike, Gang Yu, Ju Wang, Yifei Zheng

**Affiliations:** 1 School of Public Administration, China University of Geosciences, Wuhan, Hubei, China; 2 School of Public Health, Chengdu University of Traditional Chinese Medicine, Chengdu, Sichuan, China; 3 Warwick Business School, University of Warwick, Coventry, United Kingdom; 4 Liangshan Prefecture Center for Disease Control and Prevention, Xichang, Sichuan, China

**Keywords:** HIV-TB co-infection, treatment adherence, multi-disease prevention policy, healthcare workers, mixed-methods

Affiliations ^“^School of Public Health, Chengdu University of Traditional Chinese Medicine, Chengdu, Sichuan, China” and ^“^Warwick Business School, University of Warwick, Coventry, United Kingdom” were erroneously ordered incorrectly. Their numbering has changed from “3” and “2” respectively to “2” and “3” respectively.

The funder Liangshan Science and Technology Program Key R&D Project (Grant no. 23ZDYF0025) was erroneously omitted. The correct **Funding** statement reads: “The author(s) declared that financial support was received for this work and/or its publication. This work was supported by the National Natural Science Foundation of China (Grant no. 72304050) and Liangshan Science and Technology Program Key R&D Project (Grant no. 23ZDYF0025).”

The original version of this article has been updated.

